# Design and Characterization of Microscale Auxetic and Anisotropic Structures Fabricated by Multiphoton Lithography

**DOI:** 10.3390/nano11020446

**Published:** 2021-02-10

**Authors:** Ioannis Spanos, Zacharias Vangelatos, Costas Grigoropoulos, Maria Farsari

**Affiliations:** 1 Department of Engineering Science, University of Oxford, Oxford OX1 4BH, UK; spanosjo@gmail.com; 2 Nonlinear Lithography Laboratory, Institute of Electronic Structure and Laser (IESL), Foundation for Research and Technology, Hellas (FORTH), 70013 Heraklion, Crete, Greece; 3 Laser Thermal Laboratory, Department of Mechanical Engineering, University of California, Berkeley, CA 94720, USA; zacharias_vangelatos@berkeley.edu (Z.V.); cgrigoro@berkeley.edu (C.G.)

**Keywords:** mechanical metamaterials, controlled anisotropy, auxeticity, FEA simulations, multiphoton lithography, in-situ SEM-nanoindentation, mechanical characterization, architected designs

## Abstract

The need for control of the elastic properties of architected materials has been accentuated due to the advances in modelling and characterization. Among the plethora of unconventional mechanical responses, controlled anisotropy and auxeticity have been promulgated as a new avenue in bioengineering applications. This paper aims to delineate the mechanical performance of characteristic auxetic and anisotropic designs fabricated by multiphoton lithography. Through finite element analysis the distinct responses of representative topologies are conveyed. In addition, nanoindentation experiments observed in-situ through scanning electron microscopy enable the validation of the modeling and the observation of the anisotropic or auxetic phenomena. Our results herald how these categories of architected materials can be investigated at the microscale.

## 1. Introduction

Architected mechanical metamaterials are often defined as contrived materials with épatant mechanical properties. Another name for such structures is mechanical metamaterials, since they can possess features that surpass the conventional behavior observed in natural materials [1]. These properties are a reflection of their structural configuration, and not a consequence of the chemical composition of their constituent bulk materials. Characteristic paradigms of thoroughly investigated designs are remarkably strong, ultra-light materials for aerospace applications [2]; materials with negative Poisson’s ratio (auxetics) which can be used in medical implants and sports equipment [3,4]; pentamode metamaterials, i.e., solids which can regulate the wave propagation on the solid in the acoustic regime and can be used as the building blocks for materials with completely arbitrary elastic properties [5,6].

Until recently, the realization and study of mechanical metamaterials existed only in two dimensions as a consequence of the less perplexing analysis that is required in 2D elasticity, plasticity and fracture mechanics [7]. As additive manufacturing techniques became more widely available, the fabrication of mechanical metamaterials was elevated into the realm of three-dimensions, leading to several riveting and scintillating applications such as biomedical implants [8], battery electrodes [9], and scaffolds for tissue engineering [10,11]. Some of the techniques employed in the fabrication of 3D mechanical metamaterials are inkjet printing, selective laser melting, and multiphoton lithography (MPL) [12].

More specifically, the implementation of architected materials in ceramic nanolattices elucidated how such topologies can supress brittle behavior and emulate ductility under cyclic loading. This remarkable result has been demonstrated for both conventional hollow lattices [13] and the substantially more elusive woven-spiral lattices [14]. Furthermore, an emerging class of metamaterials, scilicet spinodoid metamaterials, has been investigated at the microscale due to their tailored anisotropy, rendering them a potential candidate for controlled wave propagation [15].

Auxetic metamaterials are characterised by a negative Poisson’s ratio [16,17] and have extraordinary mechanical properties such as high energy absorption and fracture resistance. They are widely found in Nature, as a large number of crystalline materials are auxetic [18,19,20]. One of the first auxetic mechanical structures was proposed by Almgren in 1985 [21]. The first auxetic foam was presented by Lakes [22], while the first 2D molecular models spontaneously forming auxetic phases were studied by Wojciechowski [23,24]. The term auxetic was first introduced in the scientific literature by Evans [25]. Since then, research on auxetic systems has increased rapidly, as applications in a wide range of fields have been proposed [26].

In general, auxetics have an interesting reaction to different types of stress. During an impact, the material is laterally compressed making it harder to penetrate [27]. Also, the energy of the impact is dissipated in the transverse directions, greatly reducing the peak stress at bottom layers [28]. When an auxetic is stretched, it expands in all directions due to its individual cells comprising it getting bigger. If a crack is formed, the cellular expansion will tend to close it, providing a fracturing resistance [20]. Therefore, auxeticity can obstruct a detrimental stress strain response that can lead to stress concentration, defect propagation and necking instability. Hence, the cyclic response of the specimen is prolonged and catastrophic failure is hindered. Consequently, auxetics are prime candidates for durable shock absorbers in body protection equipment [29]. Furthermore, it might be possible to take advantage of the negative Poisson’s ratio to increase the accuracy of piezoelectric sensors and actuators or create tunable filters with a uniform increase/decrease in pore size [30].

3D negative Poisson’s ratio structures have been proposed as auxetic stents used in opening up clogged blood vessels [31]. Once placed inside the vessel, blood flow will expand the stent along its axis, which causes it to also expand laterally, opening up the walls of the vessel. In addition, several studies have been emerged illuminating how specific tissues such as bone or tendons can possess auxetic behavior [32]. The same theme is desired in controlled anisotropy. In applications such as aerospace engineering, the enhanced directional rigidity of specific components is idoneous, while aiming to reduce the weight of the component through lattice or composite topologies.

We have fabricated our structures using multiphoton lithography (MPL). MPL is a laser-based additive manufacturing technique which provides the fabrication of 3D micro-structures with sub-micron resolution [12]. Based on the physical principle of multiphoton absorption, it can be used with a variety of photosensitive, transparent materials to make free-form 3D structures using a computer model (CAD).

In this paper, we present our work towards the construction of functional mechanical metamaterials microstructures. First, we investigate the mechanical properties of the bulk material used in MPL. Using these data, we conduct finite element analysis (FEA) simulations to evaluate the designs and control the fabrication parameters. Then, we fabricate a series of auxetic devices based on the re-entrant triangular design and characterize their composite material. Finally, we propose a new geometry which can act as a vertical displacement spring. Through in-situ scanning electron microscopy (SEM) nanoindentation experiments, we test and validate the response of the tested specimens. Our results and methodology present a design strategy that can be utilized in the modeling, fabrication and testing or microscale auxetic and anisotropic metamaterials for biomedical applications.

## 2. Materials and Methods

### 2.1. Material & Sample Preparation

The material employed for the scaffold fabrication is an organic-inorganic hybrid described previously [33]. The material preparation is as follows: zirconium propoxide (ZPO, 70% in propanol) was mixed with 2-(dimethylamino)ethyl methacrylate (DMAEMA), and the mixture was stirred for 15 min before the addition of hydrolyzed methacryloxypropyl trimethoxysilane (MAPTMS) and the photoinitiator 4,4-bis(diethylaminobenzophenone) (Michler’s ketone). After stirring for 30 min, the composite was filtered using a 0.22 micron syringe filter. The samples were prepared by drop-casting onto glass substrates and the resulting samples were dried in an oven at 100 °C for 1 h. After the printing of the structure with MPL, the samples were developed in a 70:30 solution of 1-propanol: isopropanol and further rinsed with isopropanol. All chemicals were obtained from Sigma-Aldrich (Taufkirchen, Germany).

### 2.2. 3D Structure Fabrication Using MPL

The experimental setup used in this work is the same as described in [11]. It consists of two scanning systems using the same irradiation source: a Ti:Sapphire pulsed fs laser operating at 800 nm (Femtolasers Fusion, Vienna, Austria, pulse duration <20 fs in the sample, repetition rate 80 MHz). The first setup (referred to as *nano setup*) employs piezoelectric and linear stages for the x-y-z movement (Physik Instrumente, Karlsruhe, Germany). The second setup uses a galvanometric mirror system (Scanlabs Hurryscan II, Puchheim, Germany) to scan the laser beam inside the material on the xy-plane, while a linear translation stage moves the sample in the z-direction. The galvanometric scanner was adapted to house a microscope objective lens. This will be referred to as the Galvo set-up.

### 2.3. Nano-Dynamic Mechanical Measurement and Analysis (Nano-DMA)

The mechanical properties of the employed resin after photopolymerization were characterised using a TI 950 TriboIndenter (Bruker, Eden Prairie, MN, USA ) to conduct nano-DMA experiments, a dynamic testing technique equipped with continuous measurement of X (CMX) control algorithms that provide a continuous measurement of mechanical properties as a function of indentation depth, where X can be hardness H, storage modulus E’, loss modulus E’’, complex modulus E* and the mechanical damping tanδ. The technique can be applied from ultra-soft hydrogels to hard coatings, with a greatly improved signal to noise ratio. Dynamic testing can be performed in a range of frequencies between 0.1–300 Hz. A quasi-static force, up to 10 mN, is applied to the indentation probe while superimposing a small oscillatory force of 5 mN maximum. A lock-in amplifier measures phase and amplitudes changes in the resulting force-displacement signal. To obtain the viscoelastic properties the material is modeled as two Kelvin -Voigt systems in parallel, with one end fixed and applying the indenters force to the other. This model requires four viscoelastic parameters to calculate the moduli of the material, providing highly accurate results compared to the simple Maxwell model or even the Standard Linear Solid [34]. More specifically, a Berkovich 142.3° 50 nm tip radius nanomechanical probe was utilized to apply a 5 Hz periodic load on cubic 40 × 50 × 50 μm^3^ samples of DMAEMA 10% with 10 nm amplitude to calculate the hardness, the loss modulus and the storage modulus of the viscoelastic response. Each measurement was conducted on specimens fabricated with different fluence to investigate the effect of the laser on the mechanical properties of the photoresist. For repeatability of the measurements, each specimen was tested at least 10 times on locations at least 0.5 μm away from the previously indented location to avoid the distortion of the results due to stress localization.

### 2.4. In-Situ SEM -Microindentation Experiments

To evaluate the mechanical performance of the fabricated architected structures, micromechanical measurements we conducted utilizing the nanoindentation apparatus (PI 85 SEM PicoIndenter, Hysitron, Bruker, Eden Prairie, MN, USA), positioned inside the chamber of a scanning electron microscope (Quanta 3D FEG, FEI, Hilsboro, OR, USA) for high precision measurements with extremely low noise to amplitude ratio. The mechanical measurement was recorded using the Hysitron Triboindenter software that is used to control the indenter. A flat tip (model 72SC-D3/035 (407A-M)) of 120 μm diameter was used in all the compression tests. The glass substrates on which the specimens were fabricated were fixed onto an SEM pin stub mount (TED PELLA, Redding, CA, USA) with PELCO^®^ Pro C100 Cyanoacrylat te Glue, (TED PELLA, Redding, CA, USA). Each structure was deformed at a rate of 250 nm/s to a maximum compressive strain of 10 μm and immediate unloading such that the development of viscoelastic phenomena is hampered. To validate the repeatability of the tests, at least 8 measurements were conducted on each different design. To evaluate the mechanical performance, the measured force-displacement curves were compared with the recorded deformation to distinguish any blatant variation in the mechanical performance that is reflected in the measured curves. To evaluate the Poisson ratio of the bulk material, cylinders of 70 μm height and 8 μm diameter were compressed and the transverse to axial strain ratio was measured by capturing the SEM image of the deformation.

## 3. Results

### 3.1. Nanomechanical Characterisation of the Photoresist

**Figure 1:**
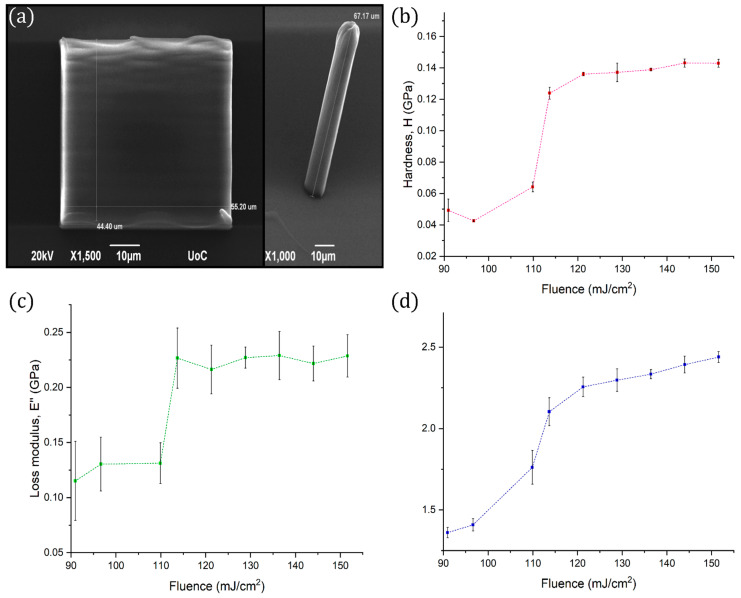
(**a**) SEM images of the cubes and the cylinders used for the evaluation of the mechanical properties, (**b**) Hardness (red line) vs. fluence, (**c**) Loss modulus (green line) vs. fluence, and (**d**) Storage modulus (blue line) vs. fluence. The abrupt change at ~110 mJ/cm^2^ is attributed to the printing strategy followed.

The mechanical properties of the photopolymerized composite were evaluated using the mechanical characterization procedures presented in the methods section to quantify its hardness H, storage modulus E’, loss modulus E’’ and Poisson’s ratio ν, as a function of the fabrication laser fluence. Arrays of cubes and cylinders were fabricated with laser fluence ranging from 90–150 (mJ/cm^2^) to evaluate the properties of the material, as it was analyzed in the previous section. All structures were fabricated with a scanning speed of 10 μm/s at the Galvo-scanner system. The measurements were conducted and analyzed utilizing nano-DMA, as it was described earlier. The cubes were used for the measurement of H, E’ and E’’, while the cylinders were used for the measurement of Poisson’s ratio, by using an additional high-speed camera to accurately capture the structures’ deformation. The plots of H, E’ and E’’ with respect to laser fluence are shown in Figure 1.

It is evident that a transition takes place at the vicinity of 110 mJ/cm^2^. As it has been discussed previously [11], this material behaviour is attributed to the polymerization threshold and the fabrication strategy which was followed. A line-by-line hatching strategy was used for each structure, with a ~200 nm distance between the centers of parallel lines. As the laser’s pulses have a repetition rate of 80 MHz and the laser beam is scanned at a 10 μm/s speed, the duration between consecutive laser pulses in the order of 1 pm. Thus, the test samples consist of densely cross-linked plates and the less dense non-polymerized material between them, which has a cross-link density dependent on the photon flux of the laser beam. Above a certain laser fluence, approximately at ~110 mJ/cm^2^ in this case, the cross-linking density saturates and can no longer increase.

In addition, the Poisson’s ratio was found to be independent of the laser fluence and have a constant value of 0.490 ± 0.002. This finding can be adduced to expound that DMAEMA 10% behaves like an incompressible material.

### 3.2. Auxetics Metamaterials

**Figure 2:**
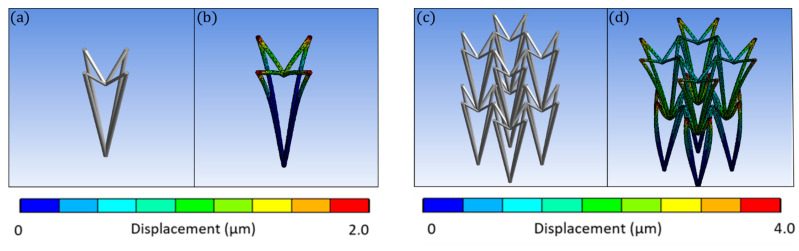
FEA simulations of the re-entrant triangular unit cell. (**a**) The undeformed unit cell, (**b**) A compression in the lateral direction causes compression of the beam members in the transverse direction, (**c**) An undeformed 2 × 2 × 2 undeformed lattice, and (**d**) Compression along the lateral direction causes a clearly more visible compression of the beam members in the transverse direction. Thus, the possession of auxetic behavior is scalable to an array of unit cells.

**Figure 3:**
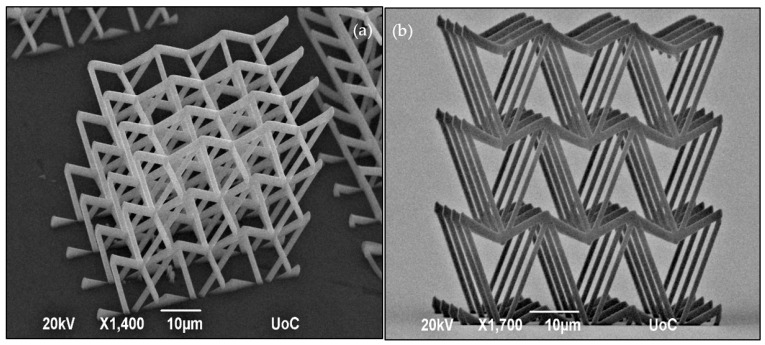
SEM images of the 2D tunable filter comprised of the re-entrant triangular fabricated using Nano-cube at 90 mJ/cm^2^ laser fluence and 100 μm/s scan speed. (**a**) Perspective view, and (**b**) Side view.

**Figure 4:**
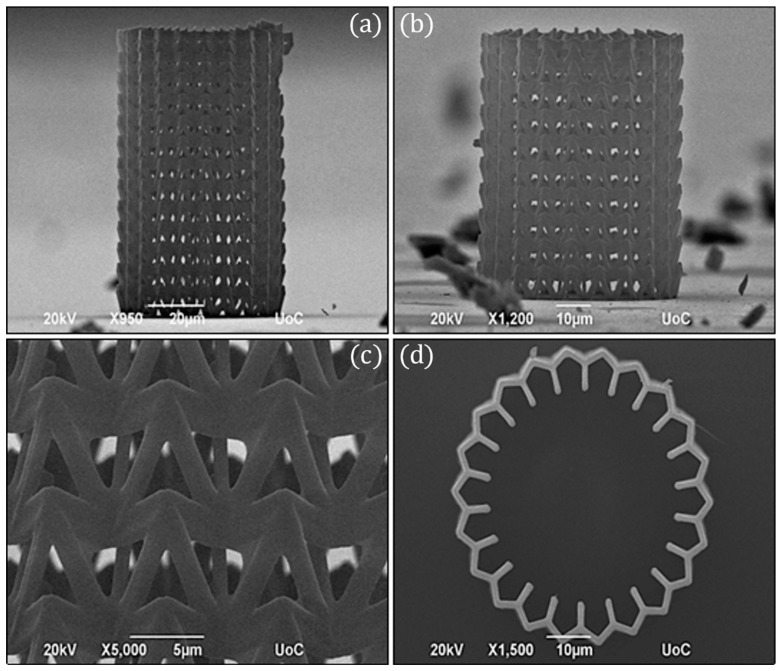
SEM images of the auxetic stent comprised of the re-entrant triangular fabricated using Nano-cube at 90 mJ/cm^2^ laser fluence and 100 μm/s scan speed. (**a**) Side view of ~90 nm in height stent, (**b**) Side view of ~60 nm in height stent, (**c**) Close view of the lattice, and (**d**) Top view.

In this study, the re-entrant triangular unit cell, a thoroughly studied auxetic geometry [35], was investigated. The behaviour of the unit cell and a 2 × 2 × 2 lattice can be seen in the FEA simulations of Figure 2. All of the simulations were modelled with Ansys R18 and conducted in the linear elastic domain, using 3D–10–node tetrahedral solid elements. Specifically, the single re-entrant triangular unit cell is comprised of 5529 nodes and 2428 elements. The array is comprised of 13,532 nodes and 4536 elements. To match the loading conditions of the experimental setup, the bottom intersection of the unit cells was fixed, while the top intersections were set to a displacement of 2.0 μm and 4.0 μm, respectively. Under axial compression, lateral compression also occurs, resulting in a negative Poisson’s ratio. By measuring the maximum strain in the lateral and transverse dimensions, the ratio is close to −1 for both cases. However, it is observed that in the array there is more excessive deformation on the middle node, resulting in a more abrupt effect at the intersection of layers. Under axial compression, lateral compression also occurs, resulting in a negative Poisson’s ratio. Fabricated strutures based on this geometry could be used as tunable filters (Figure 3) and auxetic stents (Figure 4). Both structures were realised using the Nano-Cube setup with a laser fluence of 90 mJ/cm^2^ at 100 μm/s scanning speed. The unit cell of the filter is ~10 μm and of the stent ~5 μm. The diameter of the entire stent is ~50 μm.

### 3.3. Chiral Mechanical Metamaterials

Chiral mechanical metamaterials exhibit a rotational response, perpendicular to their main axis, when an axial force is applied along it [36,37]. Properties such as this, often described as “mechanical activity” (corresponding to optical activity in optics), are the result of geometrical optimization, arising from intense and aimed computations in continuum mechanics. This degree of freedom has become accessible only in the recent years and could give rise to advanced metamaterial devices in the future. Applications could potentially include mode conversion, force field steering or dynamic mechanical cloaking [38].

**Figure 5:**
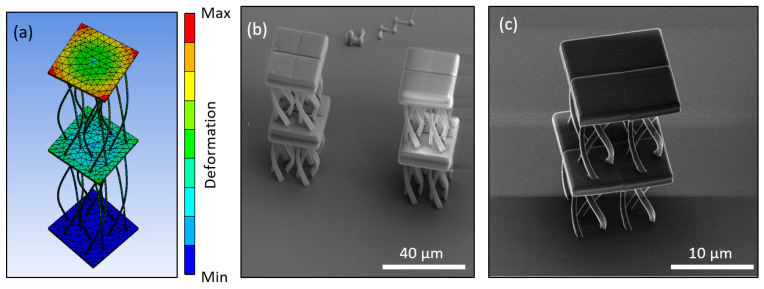
Design and fabrication of the twisting mechanical metamaterial fabricated using Nano-cube at 90 mJ/cm^2^ and 100 μm/s scan speed. (**a**) FEA simulation of the structure under loading, revealing the anisotropic response of rotation due to uniaxial loading, (**b**) Isometric view of SEM imaging of such unit cells, and (**c**) Close view of the anisotropic structure using the Helium Ion Microscope.

Numerous approaches to mechanical chirality have been presented [39]. In this work, a novel geometry with no sharp corners was fabricated. It consists of three rods that spiral around the center of the unit cell as the height changes for a total turn of 180°. In Figure 5a, a qualitative mechanical simulation of the structure is shown. Two-unit cells stacked on top of each other were modelled, so the rotation will be clearly visible as the middle cap rotates. To perform this simulation, the structure was discretized using the same element type and with 24,638 nodes and 9360 elements. The bottom base was fixed, and the top plate was subject to a 0.5 μm vertical compression. It is shown that the compression of the structure results in rotation of the unit cell. During the actual indentation measurements, the top and bottom of the structure are not free to move. The structure twists clockwise when pushed downwards. The fabricated specimens are shown in Figure 5b,c. They were made using the Nano-Cube set-up at 90 mJ/cm^2^ laser fluence and 100 μm/s scan speed.

### 3.4. Micromechanical Testing

**Figure 6:**
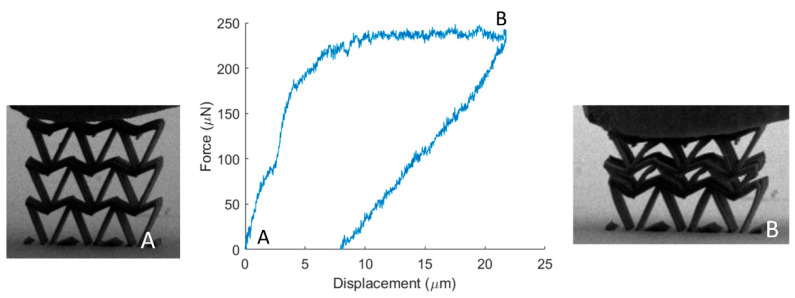
Mechanical testing of the re-entrant triangular filter. From stage **A** (undeformed configuration), the structure gets compressed, leading to the emergence of the auxetic behavior and the densification of the structure (stage **B**). A video of this mechanical testing is included Supplementary Information (filter.mp4).

**Figure 7:**
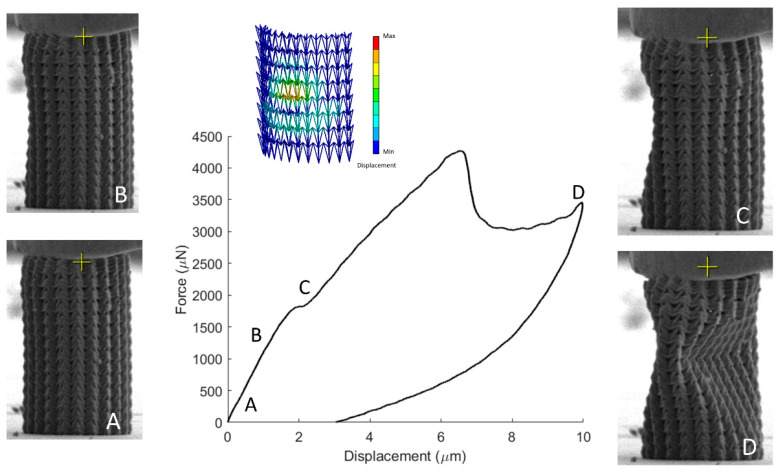
Mechanical testing of the re-entrant triangular stent. While the auxetic effect suppresses barrel shape formation, it leads to the formation of a nonlinear buckling mode which needs to be included in the design methodology. A video of this mechanical testing is included Supplementary Information (stent.mp4).

To assess the mechanical performance of the fabricated specimens, in-situ SEM microindentation experiments were conducted. A characteristic stress strain response of the re-entrant triangular is presented in Figure 6. As the structure deforms, the beam members traverse to the interior of the structure, leading to a plateau in the force-displacement curve. This deformation mode provides densification of the specimen and obstructs egregious features such as barrel shape formation, verifying the feasibility of the auxetic design. The compression of the stent-like array is presented in Figure 7. In this design, the auxetic behavior precipitates instability similar to non-linear shell buckling, as it was validated by the FEA simulations shown in Figure 7. For this simulation, a third of the structure was simulated by applying periodic boudary conditions. The structure was comprised of 53,800 nodes and 276,988 elements. To match the experiments, the bottom nodes were fixed and the top nodes were subject to a unit load. This loading condition leads to a buckling mode that demonstrates large deformation on the middle of the array, as it was also observed in the experiment. This instability lead to a drop in the force displacement curve, and contraction of the structure. For a stent mechanism that is subject to a uniform axial load on its surface, such a deformation mode must be taken into consideration, in order to obtain the correct fabrication parameters before applying it to the patient. This is an extra parameter that must be accounted for in the design of auxetic specimens and has not been highlighted before. Nevertheless, the resilience of the auxetic design to cyclic loading can countervail this additional design constraint.

**Figure 8:**
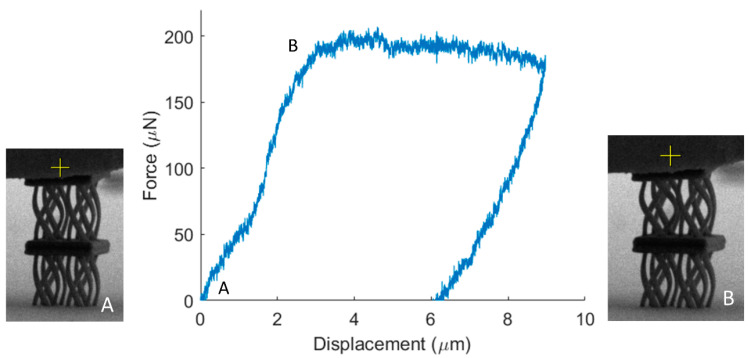
Mechanical testing of the twisting mechanical metamaterial. During the compression of the structure, the unit cell rotates leading to densification through contact of the curved members and a plateau in the response of the structure.

Furthermore, to validate the FEA simulations of the proposed anisotropic design, the fabricated twisting mechanical metamaterial was also tested (Figure 8). Again, the twist of the unit cell leads to entanglement of the undulated members, that causes the plateau in the force displacement curve for large deformations. This mechanism, apart from deformation modes similar to chirality, can improve the mechanical performance through densification, augmenting the mechanical performance beyond the elastic domain.

## 4. Discussion and Conclusions

In summary, the modelling, fabrication and testing of auxetic and anisotropic lattices structures was investigated. Utilizing FEA simulations, their intrinsic behavior was validated and modulated. Fabrication of such structures through MPL and in-situ micromechanical testing provided insight into their mechanical behavior. While auxeticity can improve the mechanical performance, specific topologies can possess additional design constraints that need to be evaluated before the structure is utilized. In addition, anisotropy converts uniaxial compression to shear due to torque can provide densification mechanisms and lead to an improved mechanical performance. These results provide a strategic approach to implement architected structures with tailored elastic properties in microscale and validate their feasibility before they are employed in bioengineering applications, such as tissue engineering or stent technologies and percolation.

Our future plans include the optimization of unit cell and structure size and future resolution, as well as the investigation of other, novel geometries with extra-ordinary properties.

## Supplementary Materials

The following are available online at https://www.mdpi.com/2079-4991/11/2/446/s1, Video S1: filter.mp4, Video S2: stent.mp4.

## References

[1] Surjadi J.U., Gao L., Du H., Li X., Xiong X., Fang N.X., Lu Y. (2019). Mechanical Metamaterials and Their Engineering Applications. Adv. Eng. Mater..

[2] Farina I., Goodall R., Hernández-Nava E., Di Filippo A., Colangelo F., Fraternali F. (2019). Design, microstructure and mechanical characterization of Ti6Al4V reinforcing elements for cement composites with fractal architecture. Mater. Des..

[3] Ren X., Das R., Tran P., Ngo T.D., Xie Y.M. (2018). Auxetic metamaterials and structures: A review. Smart Mater. Struct..

[4] Wang Z., Hu H. (2014). Auxetic materials and their potential applications in textiles. Text. Res. J..

[5] Fok L., Ambati M., Zhang X. (2008). Acoustic Metamaterials. MRS Bull..

[6] Ambati M., Fang N., Sun C., Zhang X. (2007). Surface resonant states and superlensing in acoustic metamaterials. Phys. Rev. B.

[7] Gould P.L., Feng Y. (2018). Introduction to Linear Elasticity.

[8] Nene S.S., Kashyap B., Prabhu N.V., Estrin Y., Al-Samman T. (2015). Biocorrosion and biodegradation behavior of ultralight Mg–4Li–1Ca (LC41) alloy in simulated body fluid for degradable implant applications. J. Mater. Sci..

[9] Wheeler J.P., Brill J.N., Miller L.E. (1984). Lattice for a Battery Electrode Substrate. U.S. Patent.

[10] Ma Z., Huebsch N., Koo S., Mandegar M.A., Siemons B., Boggess S., Conklin B.R., Grigoropoulos C.P., Healy K.E. (2018). Contractile deficits in engineered cardiac microtissues as a result of MYBPC3 deficiency and mechanical overload. Nat. Biomed. Eng..

[11] Flamourakis G., Spanos I., Vangelatos Z., Manganas P., Papadimitriou L., Grigoropoulos C., Ranella A., Farsari M. (2020). Laser-made 3D Auxetic Metamaterial Scaffolds for Tissue Engineering Applications. Macromol. Mater. Eng..

[12] Skliutas E., Lebedevaite M., Kabouraki E., Baldacchini T., Ostrauskaite J., Vamvakaki M., Farsari M., Juodkazis S., Malinauskas M. (2021). Polymerization mechanisms initiated by spatio-temporally confined light. Nanophotonics.

[13] Schaedler T.A., Jacobsen A.J., Torrents A., Sorensen A.E., Lian J., Greer J.R., Valdevit L., Carter W.B. (2011). Ultralight Metallic Microlattices. Science.

[14] Moestopo W.P., Mateos A.J., Fuller R.M., Greer J.R., Portela C.M. (2020). Pushing and Pulling on Ropes: Hierarchical Woven Materials. Adv. Sci..

[15] Cory H., Zach C. (2004). Wave propagation in metamaterial multi-layered structures. Microw. Opt. Technol. Lett..

[16] Saxena K.K., Das R., Calius E.P. (2016). Three Decades of Auxetics Research—Materials with Negative Poisson’s Ratio: A Review. Adv. Eng. Mater..

[17] Barchiesi E., Spagnuolo M., Placidi L. (2019). Mechanical metamaterials: A state of the art. Math. Mech. Solids.

[18] Baughman R.H., Shacklette J.M., Zakhidov A.A., Stafström S. (1998). Negative Poisson’s ratios as a common feature of cubic metals. Nature.

[19] Kimizuka H., Kaburaki H., Kogure Y. (2000). Mechanism for Negative Poisson Ratios over the *α*-β Transition of Cristobalite, SiO_2_: A Molecular-Dynamics Study. Phys. Rev. Lett..

[20] Ishibashi Y., Iwata M. (2000). A microscopic model of a negative Poisson’s ratio in some crystals. J. Phys. Soc. Jpn..

[21] Almgren R.F. (1985). An isotropic three-dimensional structure with Poisson’s ratio = −1. J. Elast..

[22] Lakes R. (1987). Foam structures with a negative Poisson’s ratio. Science.

[23] Wojciechowski K. (1987). Constant thermodynamic tension Monte Carlo studies of elastic properties of a two-dimensional system of hard cyclic hexamers. Mol. Phys..

[24] Wojciechowski K. (1989). Two-dimensional isotropic system with a negative poisson ratio. Phys. Lett. A.

[25] Evans K.E. (1991). Auxetic polymers: A new range of materials. Endeavour.

[26] Tretiakov K.V., Wojciechowski K.W. (2020). Auxetic, Partially Auxetic, and Nonauxetic Behaviour in 2D Crystals of Hard Cyclic Tetramers. Phys. Status Solidi (RRL)—Rapid Res. Lett..

[27] Acharyya K., Bhattacharyya S., Sepehrpour H., Chakraborty S., Lu S., Shi B., Li X., Mukherjee P.S., Stang P. (2019). Self-Assembled Fluorescent Pt(II) Metallacycles as Artificial Light-Harvesting Systems. J. Am. Chem. Soc..

[28] Yang C., Vora H.D., Chang Y. (2018). Behavior of auxetic structures under compression and impact forces. Smart Mater. Struct..

[29] Bezazi A., Boukharouba W., Scarpa F. (2009). Mechanical properties of auxetic carbon/epoxy composites: Static and cyclic fatigue behaviour. Phys. Status Solidi.

[30] Alderson A., Rasburn J., Ameer-Beg S., Mullarkey P.G., Perrie W., Evans K.E. (2000). An Auxetic Filter: A Tuneable Filter Displaying Enhanced Size Selectivity or Defouling Properties. Ind. Eng. Chem. Res..

[31] Li X., Wang Q., Yang Z., Lu Z. (2019). Novel auxetic structures with enhanced mechanical properties. Extrem. Mech. Lett..

[32] Yao Y., Wang L., Li J., Tian S., Zhang M., Fan Y. (2020). A novel auxetic structure based bone screw design: Tensile mechanical characterization and pullout fixation strength evaluation. Mater. Des..

[33] Danilevicius P., Rezende R.A., Pereira F.D.A.S., Selimis A., Kasyanov V., Noritomi P.Y., Da Silva J.V.L., Chatzinikolaidou M., Farsari M., Mironov V. (2015). Burr-like, laser-made 3D microscaffolds for tissue spheroid encagement. Biointerphases.

[34] Findley W.N., Lai J.S., Onaran K. (1976). Creep and Relaxation of Nonlinear Viscoelastic Materials, with an Introduction to Linear Viscoelasticity.

[35] Yang L., Harrysson O., West H., Cormier D. (2015). Mechanical properties of 3D re-entrant honeycomb auxetic structures realized via additive manufacturing. Int. J. Solids Struct..

[36] Frenzel T., Kadic M., Wegener M. (2017). Three-dimensional mechanical metamaterials with a twist. Science.

[37] Reinbold J., Frenzel T., Münchinger A., Wegener M. (2019). The Rise of (Chiral) 3D Mechanical Metamaterials. Materials.

[38] Milton G.W., Briane M., Willis J.R. (2006). On cloaking for elasticity and physical equations with a transformation invariant form. New J. Phys..

[39] Jenett B., Cameron C., Tourlomousis F., Rubio A.P., Ochalek M., Gershenfeld N. (2020). Discretely assembled mechanical metamaterials. Sci. Adv..

